# Mature leaf concentrate of Sri Lankan wild type *Carica papaya* Linn. modulates nonfunctional and functional immune responses of rats

**DOI:** 10.1186/s12906-017-1742-z

**Published:** 2017-04-26

**Authors:** Chanika Dilumi Jayasinghe, Dinara S Gunasekera, Nuwan De Silva, Kithmini Kawya Mandakini Jayawardena, Preethi Vidya Udagama

**Affiliations:** 1 Department of Zoology & Environment Sciences, Faculty of Science, University of Colombo, Colombo, 3 Sri Lanka; 2 Sri Lanka Institute of Nanotechnology, Mahenwatte, Pitipana, Homagama, Sri Lanka; 3 Department of Biotechnology, Faculty of Agriculture & Plantation Management, Wayamba University of Sri Lanka, Wayamba, Sri Lanka

**Keywords:** *Carica papaya*, Sri Lankan wild type cultivar, Mature leaf concentrate, Immunomodulation, Immune cell counts, Phagocytosis, Cytokines

## Abstract

**Background:**

The leaf concentrate of *Carica papaya* is a traditionally acclaimed immunomodulatory remedy against numerous diseases; nonetheless comprehensive scientific validation of this claim is limited. The present study thus investigated the immunomodulatory potential of *Carica papaya* mature leaf concentrate (MLCC) of the Sri Lankan wild type cultivar using nonfunctional and functional immunological assays.

**Methods:**

Wistar rats (*N* = 6/ group) were orally gavaged with 3 doses (0.18, 0.36 and 0.72 ml/100g body weight) of the MLCC once daily for 3 consecutive days. Selected nonfunctional (enumeration of immune cells and cytokine levels) and functional (cell proliferation and phagocytic activity) immunological parameters, and acute toxic effects were determined using standard methods.

Effect of the MLCC (31.25, 62.5, 125, 250, 500 and 1000 μg/ml) on ex vivo proliferation of bone marrow cells (BMC) and splenocytes (SC), and in vitro phagocytic activity of peritoneal macrophages (PMs), and their corresponding cytokine responses were evaluated. The phytochemical profile of the MLCC was established using liquid chromatography-mass spectrometry (LS-MS) and Gas chromatography-mass spectrometry (GC-MS).

**Results:**

Counts of rat platelets, total leukocytes, lymphocyte and monocyte sub populations, and BMCs were significantly augmented by oral gavage of the MLCC (*p* < 0.05). The highest MLCC dose tested herein significantly reduced pro inflammatory cytokines, Interleukin 6 (IL-6) and Tumor Necrosis Factor α (TNF α) levels of rats (*p* < 0.05).

The in vivo phagocytic index of rat PMs significantly increased by oral gavage of all three doses of the MLCC (*p* < 0.05). In vitro phagocytic activity of rat PMs were enhanced by the MLCC and triggered a Th1 biased cytokine response.

The MLCC at low concentrations elicited ex vivo proliferation of BMC (31.25 μg/ml) and SC (31.25 and 62.5 μg/ml) respectively. Conversely, high concentrations (500 and 1000 μg/ml) exhibited cytotoxicity of both BMC and SC with significant modulation of cytokines. Chemical profile of the MLCC revealed the presence of several immunomodulatory compounds. The oral gavage of the MLCC was found to be safe in terms of both hepatic and renal toxicities.

**Conclusion:**

The present study established that the mature leaf concentrate (MLCC) of *Carica papaya* Sri Lankan wild type cultivar is orally active, safe and effectively modulates nonfunctional and functional immunological parameters of rats that unequivocally corroborate the traditional medical claims.

## Background

Pharmacological manipulation of the immune system is emerging as a novel approach in managing human diseases. There is a recent spurt in the interest of identification of immunomodulatory leads of herbal origin which are safe, efficient and economical in chronic usage.

The immune system is an ensemble of complex defense mechanisms functioning at different levels, from an individual cell to the whole organism [1]. Hence, imbalance of the immune homeostasis is implicated in the onset and progression of chronic ailments [2]. An overactive immune system is associated with autoimmunity, chronic inflammatory diseases, systemic vasodilatation and carcinogenesis [3, 4].Conversely, immunosupression increases the susceptibility to infection and is implicated in tumor development [5, 6].

The clinical concept of immunomodulation focuses on stimulating both innate and adaptive arms of immunity or suppressing excessive immune function using exogenous therapeutic agents [2]. Synthetic cytotoxic drugs (cyclophosphamide, methotrexate and 5-fluorouracil), recombinant cytokine therapy, and antibody therapy are established in allopathic medicine as immunotherapy against cancers and autoimmune diseases [7]. However, the occurrence of adverse events and high costs of these have generated barriers to successful therapeutic applications [8]. In this regard, plant based immunomodulators are advantageous considering their pleotropic activity, less side effects and cost effectiveness.

Since ancient times, medicinal plants have been used virtually in all cultures as sources of medicines to improve the immune system. Recently, advancements in immunological techniques enabled the characterization of medicinal plants in unprecedented detail. Numerous plant species such as *Aloe vera, Withania somnifera, Allium sativum*, *Ocimum sanctum*, *Azadirachita indica* and many others have been scientifically justified for their immunomodulatory activity together with possible mechanistics [9].

*Carica papaya*, (Common name- paw paw) belongs to family Caricaceae and is one of the most popular and economically important plants in the world as a food source and as a herbal medicine [10]. It is native to the tropics of the Americas but now is cultivated worldwide as a fruit crop [10].

Different parts of *C. papaya* has been traditionally claimed as therapeutics for a broad range of diseases [10]. Particularly the leaf extract/concentrate has been scientifically investigated for pharmacological properties such as anti-microbial, anti-parasitic (antimalarial) activity, anti-cancer, anti-inflammatory and membrane stabilization properties [10].

In Sri Lankan traditional medicine the leaf juice of *C. papaya* is claimed as a powerful remedy for modulating the immune system (Personal communication- Dr. Pathum Jayaweera, Sri Lankan traditional medical practitioner). Thus far several in vitro and in vivo experiments have been carried out to investigate the immuno pharmacological properties of the leaf concentrate of *C. papaya*.

Our group for the first time, established the platelet and total white blood cell (WBC) increasing activity of the mature leaf concentrate of *C. papaya* of the red lady cultivar grown in Sri Lanka using hydorxyurea induced thrombocytopenic Wistar rat model as well as in normal counterpart rats [11]. Concurrently, we observed pronounced anti-inflammatory activity of this preparation indicating immunomodulatory potential [11]. Similarly, oral gavage of papaya leaf extract exhibited a significant platelet and WBC increasing activity in immunosuppressive rats using cyclophosphamide [12] and ansgrelide (thrombocytopenic drug) [13].

Dharmarathna et al. [14] observed a marked platelet count elevation in none thrombocytopenic mice following the oral treatment of *C. papaya* extract for seven days [14]. Another study conducted using non thrombocytopenic rats reported a marked increment in platelet counts, mean cell haemoglobin (MCH) and mean corpuscular volume (MCV) following the oral treatment with this leaf formulation for 7 days [15].

In addition to animal experimentation, the platelet increasing activity of papaya leaf extract has been validated in thrombocytopenia associated dengue patients. An open labeled randomized controlled trial carried out with 228 patients (Treatment = 111, Control = 117) with dengue fever (DF) and dengue haemorrhagic fever (DHF) revealed a significant increase of platelet counts after 40 and 48 h of oral administration of *C. papaya* leaf juice for 3 consecutive days [16]. A pilot study reported an increase of platelet and WBC counts of dengue patients after 24 h of administration of papaya leaf juice and patients recovered without hospital admission [17]. A similar effect was also observed in an open labeled randomized controlled trial with 30 dengue patients treated with a tablet (Caripill) prepared from *C. papaya* leaf extract [18]; A significant increase of platelet counts was observed following oral treatment with this tablet three times daily for five days and exerted fewer side effects and good tolerability.

Although, a wealth of information is available on platelet increasing activity of different extracts of *C. papaya* leaves, a comprehensive account on immunomodulatory potential is limited. Otsuki et al. [19] reported immunomodulatory potential of the aqueous extract of *C. papaya* leaf using in vitro culture of human peripheral blood mononuclear cells (PBMCs).

When PBMCs were treated in vitro with *C. papaya* aqueous extract, Interleukin 2 and 4 (IL-2 and IL-4) secretion were attenuated whereas that of Interleukin 12p40, 12p70 (IL- 12p40, IL-12p70), Interferon Gamma (IFN-γ) and Tumor Necrosis Factor alpha (TNF-α) was enhanced without growth inhibition [19]. Moreover, microarray analyses showed the expression of 23 immunomodulatory genes of PBMC [20].

Tomar et al. [21] reported oral administration of alcoholic extract of papaya leaf (50–200 mg/kg) had stimulated both innate and humoral immune responses of Bagg Albino laboratory bred (BALB/c) mice.

Although, the immunomodulatory potential of different *C. papaya* extracts (aqueous and alcohol) was adequately demonstrated in the above in vitro and in vivo studies, a comprehensive account of the effect of the mature leaf concentrate of *C. papaya* leaf both on nonfunctional and functional immune responses may strongly warrant the development of a potential drug lead.

Thus, the present study specifically aimed at investigating the effect of MLCC on nonfunctional (immune cell counts and cytokine levels) and functional (cell proliferation and phagocytosis) immunological parameters of rats. Further, liver and kidney function parameters were examined to envisage the safety of oral administration of the MLCC. Moreover, the chemical profile of the MLCC was investigated using liquid chromatography-mass spectrometry (LC-MS) and Gas chromatography-mass spectrometry (GC-MS).

A preliminary study on platelet increasing activity revealed that the Sri Lankan wild type *C. papaya* variety possessed more potent platelet increasing activity than the hybrid cultivars such as red lady (unpublished data). Thus, leaves of the Sri Lankan wild type cultivar of *C. papaya* were used in this study.

## Methods

### Chemicals and reagents

Pyrosate Limulus amebocyte lysate (LAL) endotoxin determination kit was purchased from Associates for Cape Code Incorporated (E.Saint Jean Drive.E.Falmouth, MA, 02536 USA). Sandwich, Enzyme Linked Immunosorbent Assay (ELISA) kits to assess rat Interleukin 6 (IL-6) and 10 (IL-10), Interferon gamma (IFNγ) and Tumor Necrosis Factor alpha (TNF-α) were purchased from BD Bio sciences (Torreyana Rd., San Diyego, 92,121, CA). The cell culture medium and reagents were purchased from different sources, Roswell Park Memorial Institute (RMPI) 1640, from Gibco BRL Life Technologies (Grand Island, NY, USA) Fetal bovine serum (FBS), streptomycine-penicilline antibiotics, cell culture grade sodium biocarobonate, dimethyl sulfoxide (DMSO), 4,5-dimethylthiazole-2-yl)-2,5-diphenyltetrazolium bromide (MTT), Nitro blue tetrazolium (NBT) were purchased from Sigma Aldrich Co., Ltd. (St. Louis, MO, USA).

LC-MS grade methanol, formic acid and common commercial grade chemicals, ethanol, isopropanol, hydrochloric acid, ammonium oxalate, acetic acid were purchased from Sigma Aldrich Co. Ltd. (St. Louis, MO, USA). For LC-MS analysis water was purified by a Milli-Q purification system from Evoqua Water Technologies.

### Collection of plant material

Mature leaves (5th leaf from the apex) of *Carica papaya (Local name: Sinhala: papol, gaslabu; Tamil: pappali, pappayi; English: Paw paw, Papaya)* of the Sri Lankan wild type cultivar were collected from a home garden in Kadawatha, Gampaha district in Sri Lanka (longitude-79°57′0″E,latitude- 7^°^4′0″N) during April 2014 to June 2015. The specimen was identified and authenticated by Dr. H Kathriarachchi of the Department of Plant Sciences of the University of Colombo, Sri Lanka. A voucher specimen (No- 120) was deposited at the Department of Zoology and Environment Sciences, University of Colombo, Sri Lanka.

### Preparation of mature leaf concentrate

The mature leaf concentrate of *C. papaya* (MLCC) was prepared essentially following the procedure described in [11] with slight modifications. Briefly, fresh mature leaves of *C. papaya* (wild type cultivar) were thoroughly washed under running tap water, blotted dried and after removal of petioles and primary veins, leaf blades were pulverized using a mechanical juice extractor (HR,1861, Philips, Hong Kong) without adding water (at 10 g leaf blade/ ml of concentrate). The test animals were orally gavaged directly with the fresh MLCC at doses of 0.18, 0.36 and 0.72 ml/100g of body weight (BW) of rats representing low, mid (human equivalent) and high doses, respectively [11]. For the in vitro assays, the extract was filtered (0.22 μm Millipore filter, 290 Concord Rd, Billerica, MA, USA) and concentrations were calculated by extrapolating the dry weight of the MLCC.

### Detection of endotoxins

The fresh MLCC was screened for endotoxin contamination using the Limulus Amebocyte Lystate (LAL) gel-clot test according to the manufactures instructions (the Pyrosate® Kit, Associates for Cape Code Incorporated,124 Bernard E.Saint Jean Drive.E.Falmouth, MA,02536 USA).

### Experimental animals

Healthy, adult male and female Wistar rats (180–230 g of weight) purchased from the Medical Research Institute, Colombo, Sri Lanka were used in this study. All animals received humane care. They were housed in plastic cages in the animal house of the Department of Zoology and Environment Sciences, University of Colombo under standard animal house conditions (temperature; 28–31 °C, photoperiod; approximately 12 h natural light per day, relative humidity: 50–55%). The animals were fed with pelleted food (VET HOUSE Ltd. Colombo, Sri Lanka) and clear drinking water *ad libitum*. Ethical approval for the laboratory animal study was obtained from the Institute of Biology, Sri Lanka (Ethical approval number -IOBSL 111 05 29). Hence, all experiments conducted were in compliance with the Organization for Economic Co-operation and Development (OECD) guidelines.

### Effects of the MLCC on nonfunctional immunological parameters of rats

#### Enumeration of immune cells

Four separate groups of adult male Wistar rats (*N* = 6/ group) were orally gavaged with distilled water (DW) as the control, three doses of the MLCC at 0.18, 0.36 and 0.72 ml/100g BW of rats, once daily for three consecutive days. On day 3 post treatment, rats were anesthetized under high dose of ether and blood was collected by cardiac puncture and dispensed into ethylenediaminetetra acetic acid (EDTA) containing tubes. Platelets, and total and differential white blood cell counts were established according to [22]. Femur and spleen were aseptically excised from sacrificed rats and placed in glass vials containing 5 ml of phosphate buffered saline (PBS). Spleen was macerated in PBS to release the splenocytes (SC), and bone marrow cells (BMC) were separated by flushing PBS through the femur; SC and BMC counts were made using a Neubauer’s improved Haemocytometer (B.S 748, Weiber, England) [22]***.***

#### Plasma cytokine levels

Two separate groups of rats (*N* = 6/group) were orally administrated with the highest dose (0.72 ml/100g BW) of the MLCC and DW as the control once daily for three consecutive days. On day 3 post treatment, blood was collected by heart puncture and dispensed into EDTA tubes. Anticoagulated blood was immediately centrifuged at 750 x g for 10 min to separate plasma [22]. The plasma cytokine levels of IL-6, TNF-α, IFN-γ and IL-10 in both treated and control groups were quantified using rat sandwich ELISA kits according to the manufacturer’s instructions (BD Bio science, Torreyana Rd., San Diyego, CA 92121).

### Effects of the MLCC on functional immunological parameters of rats

#### Neutral red dye uptake assay

Functional immunological test based on phagocytic activity was measured using neutral red dye uptake assay [22]. In brief, peritoneal macrophages (PMs) were aspirated from the rats treated with the three doses of the MLCC (low: 0.18 ml, mid: 0.36 ml and high: 0.72 ml/100 g BW) into 10 ml of PBS [22]. The cell suspension was centrifuged at 500 x g for 5 min at RT and the resultant cell pellet was dissolved in 1 ml of PBS. Two drops of 1% Neutral Red (in PBS) were added to the cell suspension and PMs with ingested red dye particles were counted using a Neubauer imporved haemocytometer (B.S 748, Weiber, England). The phagocytic index was calculated as follows [22]:

$$ \mathrm{Phagocytic}\kern0.3em \mathrm{index}=\frac{\mathrm{Number}\kern0.3em \mathrm{of}\kern0.3em \mathrm{active}\kern0.3em \mathrm{PM}\kern0.3em \mathrm{cells}}{\mathrm{Number}\kern0.3em \mathrm{of}\kern0.5em \mathrm{total}\kern0.3em \mathrm{PM}\kern0.3em \mathrm{cells}}\times 100\% $$

### In vitro phagocytic activity and cytokine profile of rat peritoneal macrophages

#### Preparation of rat peritoneal macrophages (PMs)

Five milliters of FBS was injected intraperitoneally into rats as a stimulant to elicit PMs [23]. Three days later, the peritoneal exudate was collected by peritoneal lavage with 10 ml of complete RPMI 1640 (CRPMI) medium supplemented with 50 μM 2-mercapthoethanol.

#### NBT dye reduction assay

In vitro phagocytic activity of rat immune cells was established by the nitrobluetetrazolium (NBT) reduction assay [23]. Twenty microliters of complete RPMI 1640 (CRPMI; Control), different concentrations of the MLCC (31.25, 62.5, 125,250, 500 and 1000 μg/ml), 20 μl of the PM suspension and 40 μl of CRPMI medium were added to wells of a 96-well plate (Corning, Sigma,USA). After incubation for 24 h at 37 °C in 5% CO_2_ humidified atmosphere, 20 μl of a heat inactivated yeast (*Saccharomyces cerevisiae*) suspension (5 × 10^7^ particles/ml) and 20 μl of 1.5 mg/ml NBT in PBS were dispensed and the mixture was further incubated under the same conditions.

After incubation for 60 min, the adherent cells were rinsed vigorously with CRPMI medium and washed four times with 200 μl methanol. After air-drying, 120 μl of 2 M potassium hydroxide (KOH) and 140 μl of DMSO was added. The absorbance was measured at 570 nm using a micro plate reader (Microplate reader 680, Bio-Rad, USA) and the percentage of NBT reduction representing phagocytic activity was calculated using the following equation [23]:

$$ \mathrm{Phagocytic}\ \mathrm{activity}\ \left(\%\right)=\frac{\left[\mathrm{OD}\ \mathrm{sample}\hbox{-} \mathrm{OD}\ \mathrm{negative}\ \mathrm{control}\right]}{\mathrm{OD}\ \mathrm{negative}\ \mathrm{control}}\times 100 $$

#### Cytokine profiling of PM cell cultures

Briefly, 40 μl CRPMI (Control), different concentrations of the MLCC (31.25, 62.5, 125,250, 500 and 1000 μg/ml), 20 μl of the PM suspension and 40 μl of CRPMI medium were cultured in wells of a flat bottom 96-well plate (Corning, Sigma, USA). After incubation for 24 h at 37 °C in 5% CO_2_ humidified atmosphere, 20 μl of a heat inactivated yeast (*Saccharomyces cerevisiae*) suspension (5 × 10^7^ particles/ml) and 40 μl of CRPMI were added and incubated at the same condition for another 1 h. Cultures were centrifuged at 1000 x g at 4 °C for 10 min and the resultant supernatants were analysed for IFN-γ and IL-10 using rat sandwich ELISA kits according to the manufacturer’s instructions (BD Bio science, Torreyana Rd., San Diyego, CA 92121).Viable cells of each concentration were calculated using trypan blue dye exclusion assay [20] and cytokine levels were normalized to the viable cell count.

### Ex vivo proliferation and cytokine profiling of rat immune cells

#### Preparation of rat bone marrow cells (BMC) and splenocytes (SC)

Femurs and spleens were aseptically excised from scarified Wistar rats. Bone marrow cells were obtained by flushing PBS through the femur bone cavity [22]. Spleens were collected into PBS and the SCs were gently released. Both cell suspensions [22] were centrifuged at 300 x g, at 25 °C for 10 min [24]. The erythrocytes in the cell suspensions were then lysed by hypotonic solution (0.2% NaCl) and the cells were resuspended in 1.6% NaCl to restore the isotonicity [23]. Cell suspensions were washed twice with CRPMI medium supplemented with 10% heat-inactivated FBS, 100 U penicillin, and 100 μg/l streptomycin [23, 24]. The cell numbers were adjusted to 10^6^ cells/ml using a Neubauer’s improved haematocytometer (B.S 748, Weiber, England). Trypan blue dye exclusion assay was performed to assess the viability of both cell types [20].

#### MTT based cell proliferation assay

The in vitro cell proliferation assay was carried out using 4,5-dimethylthiazole-2-yl)-2,5-diphenyltetrazolium bromide (MTT) assay without mitogenic stimulation [24, 25]. Briefly, 20 μl of CRPMI as the control and various concentrations of the MLCC (31.25, 62.5, 125,250, 500 and 1000 μg/ml) and 2 μg/ml Lipopolysaccharides (LPS) as a positive control were added to 20 μl of BMC or SC cell suspensions (10^6^ cells/ml). Another 40 μl of CRPMI was added to the 96- well plate (Corning, Sigma,USA) and incubated at 37 °C in a humidified 5% CO_2_ atmosphere for 48 h. Subsequently, 20 μl of MTT (5 mg/ml) in PBS and 40 μl of RPMI was added. The culture medium was removed by aspiration and 100 μl of 0.04 M hydrochloride acid (HCl) in isopropyl alcohol were added to lyse the cells. Following the addition of 100 μl of distilled water absorbance was measured at 570 nm using a micro plate reader (Microplate reader 680, Bio-Rad, USA) and the % cell proliferation was calculated using the following equation [23, 24]:

$$ \mathrm{Cell}\ \mathrm{proliferation}\ \left(\%\right)=\frac{\left[\mathrm{OD}\ \mathrm{of}\ \mathrm{treated}\hbox{-} \mathrm{OD}\ \mathrm{of}\ \mathrm{control}\right]}{\mathrm{OD}\ \mathrm{of}\ \mathrm{control}}\times 100 $$

#### Cytokine profiling of rat BMC and SC cultures

Briefly, 40 μl of BMC or SC suspension was cultured with 40 μl of CRPMI (control) and different concentrations of the MLCC (31.25, 62.5, 125, 250,500 and 1000 μg/ml) and 120 μl of CRPMI in a 96 well plate for 48 h. Culture medium of each well was collected, centrifuged at 1000 x g at 4 °C for 10 min and the supernatants were subjected to cytokine analyses. Cytokine levels were quantified using rat sandwich ELISA kits according to the manufacture’s instructions (BD Bio science, Torreyana Rd., San Diyego, CA 92121). Viable cells of each concentration were calculated using trypan blue dye exclusion assay [20] and cytokine levels were normalized to the viable cell count.

### Acute toxicity of the MLCC on rats

Hepato and nephro toxic parameters were measured in rats (*n* = 6) treated for three consecutive days with the highest dose (0.72 ml/100g BW) of the MLCC compared with the control group. Serum parameters such as aspartate transaminase (AST), alanine transaminase (ALT), urea, blood urea nitrogen (BUN), creatinine levels were determined using Randox kits (Randox Laboratories Ltd., Co. Antrium, U.K) and the spectrophotometer (JASCO V560, Jasco Corporation, Tokyo, Japan) as per manufacturer’s instructions [26]**.**

### Chemical profiling of the MLCC

#### LC-MS analysis

LC-MS analysis were performed on a Waters e2695 instrument equipped with a photodiode array (PDA) detector (model 2998,). The separations were carried out on a reversed phase column (Waters Spherisorb 5 μm ODS2, 4.6x250mm) maintained at 20 °C. The sample was scanned from 200 to 800 nm with a resolution of 1.2 nm.

The mobile phase consisted of 50% of solvent A (0.1% *v*/v of formic acid in water) and 50% of solvent B (0.1% *v*/v of formic acid in methanol) at a flow rate of 500 μl/min for 30 min under isocratic conditions.

#### GC-MS analysis

GC-MS studies were carried out using Agilent 7890B GC system and MS of 5977A Mass Selective Detector (MSD). The separations were carried out on Agilent HP-5 ms ultra inert column (30 m × 250 μm × 0.25 μm). Initial temperature for the GC analysis was 50 °C (held for 5 min), which was raised to 230 °C (ramp 20 °C/min). The 230 °C temperature was maintained for 10 min. The sample(1 µl) was injected using split-less mode with an inlet temperature of 260 °C. Helium was used as the carrier gas with a flow rate of 2 ml/min. MS transfer line temperature was maintained at 260 °C. The assignment of the peaks is based on the National Institute of Standards and Technology (NIST) reference database.

### Statistical analyses

Data were expressed as Mean ± SEM (Standard error of means). The in vitro experiments were examined in three individual experiments, performed in triplicate for each concentration. One-way Analysis of variance (ANOVA) followed by post-hoc Tukey HSD Calculator were used for multiple comparison.

In vivo experiments entailed 6 rats per group and statistical comparisons were determined using the Mann Whitney U test.

SPSS −20 (IBM, US) statistical package was used for data analysis and*p < 0.05 and **p < 0.01 was considered as indicative of significance as compared to the control group.

## Results

### Detection of endotoxin contamination of the MLCC

Adding the LAL regents to the MLCC did not result in a firm clot which was visible in the positive control of the text kit indicative of absence of the endotoxins.

### Effect of the MLCC on nonfunctional immunological parameters of rats

#### Enumeration of immune cells; platelet, white blood cell (WBC), bone marrow cell (BMC) and splenocyte (SC) counts of rats

**Fig. 1:**
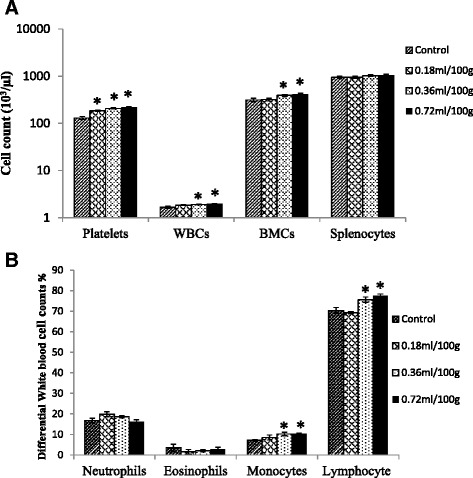
Effect of the MLCC on **a** Enumeration of rat immune cells: platelets, total white blood cells, bone marrow cells, and splenocytes **b** Differential counts of rat peripheral leukocytes. Results are expressed as Mean ± SEM for the number of animals used (*n* = 6). *p < 0.05 were considered significant when compared with the control (Mann Whitney U test)

Compared with the control, platelet counts were significantly increased by 42, 59 and 68% in rats treated with low, mid and high doses of the MLCC (*p* < 0.05), respectively (Fig. 1a). The mid and high doses of the MLCC significantly increased the total WBC by 15 and 19% (*p* < 0.05), respectively (Fig. 1a). Similarly, BMC counts significantly increased by the mid (25%) and high doses (49%) (*p* < 0.05) (Fig. 1a). Differential WBC count of the MLCC treated rats recorded a marked amelioration of monocyte and lymphocyte counts compared with the control (*p* < 0.05); Monocyte and lymphocyte counts were significantly increased by mid (43.23%, 7.5%) and high (44.67%, 10%) doses (*p* < 0.05), respectively, of the MLCC (Fig. 1b).

#### Plasma cytokine levels

**Fig. 2:**
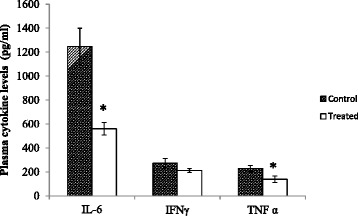
Plasma cytokines - IFNγ, TNFα, IL-6 and IL-10 levels of rats orally gavaged with the MLCC. Results are expressed as Mean ± SEM for the number of animals used (*n* = 6/group). *p < 0.05 were considered significant when compared with the control (Mann Whitney U test)

The oral administration of the highest dose of the MLCC compared with the control significantly reduced pro inflammatory cytokines, TNFα (by 39.09%) and IL-6 (by 55.06%) as depicted in Fig. 2 (*p* < 0.05). Although IFNγ levels were also reduced in treated groups compared to controls, the reduction was not significant (*p* > 0.05) (Figure 2). Plasma levels of IL-10 of both control and treated rats were below the detection level of the ELISA kit used.

### Effect of the MLCC on functional immunological parameters of rats

#### Phagocytic activity of peritoneal macrophages (PM) of rats

**Fig. 3:**
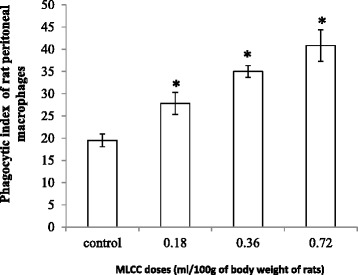
Phagocytic activity of rat peritoneal macrophages following oral treatment of MLCC. Results are expressed as Mean ± SEM for the number of animals used (*n* = 6/group). *p < 0.05 were considered significant when compared with the control (Mann Whitney U test)

The functional assay based on phagocytic activity was significantly and dose dependently (r^2^ = 0.99) increased in rats of all treated doses (low: by 43% mid: 79% and high: 109%) compared with the rats of the control group (*p* < 0.05) (Figure 3).

#### In vitro phagocytic activity of rat peritoneal macrophages (PMs)

**Fig. 4:**
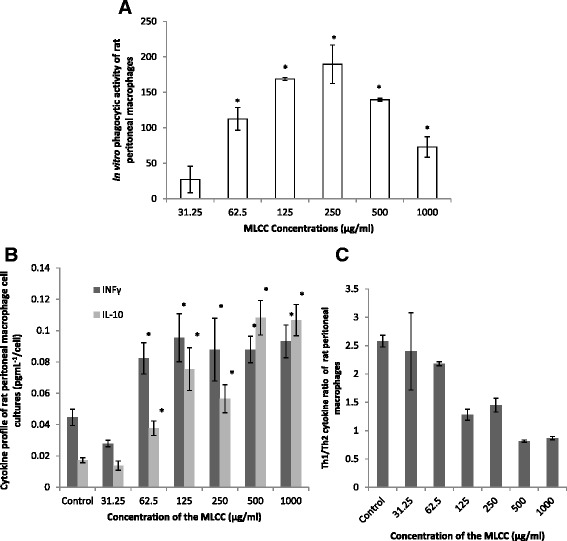
Effect of the MLCC on **a** in vitro phagocytic activity of rat peritoneal macrophages, **b** the associated cytokine; IFNγ and IL-10 profile. **c** Th1/Th2 bias cytokine response. Results are expressed as Mean ± SEM. *p < 0.05, **p < 0.01 were considered significant when compared with the control (ANOVA)

As represented in Fig. 4A the MLCC markedly enhanced in vitro phagocytic activity of rat PMs at MLCC concentrations of 62.5, 125,250, 500 and 1000 μg/ml by 112.5, 168.75, 189.58,139.5 and 72.91%, respectively (*p <* 0.05). The exception was the lowest concentration of MLCC tested (31.25 mg/ml) that had no effect on the phagocytic activity of PMs.

#### Cytokine profile of peritoneal macrophage (PM) culture

Compared with the control, the MLCC at 62.5, 125, 250, 500 and 1000 μg/ml concentrations significantly enhanced both IFNγ and IL-10 from cells in PM culture (*p* < 0.05) (Fig. 4b). However, the control and 31.25, 62.5, 125 and 250 μg/ml of MLCC exhibited a Th1 (T helper 1) biased cytokine response (Th1/Th2 > 1) while higher concentrations 500 and 1000 μg/ml concentrations exerted a Th2 biased response (Th1/Th2 < 1) (Fig. 4c).

#### Ex vivo proliferation of rat BMCs and SCs

**Fig. 5:**
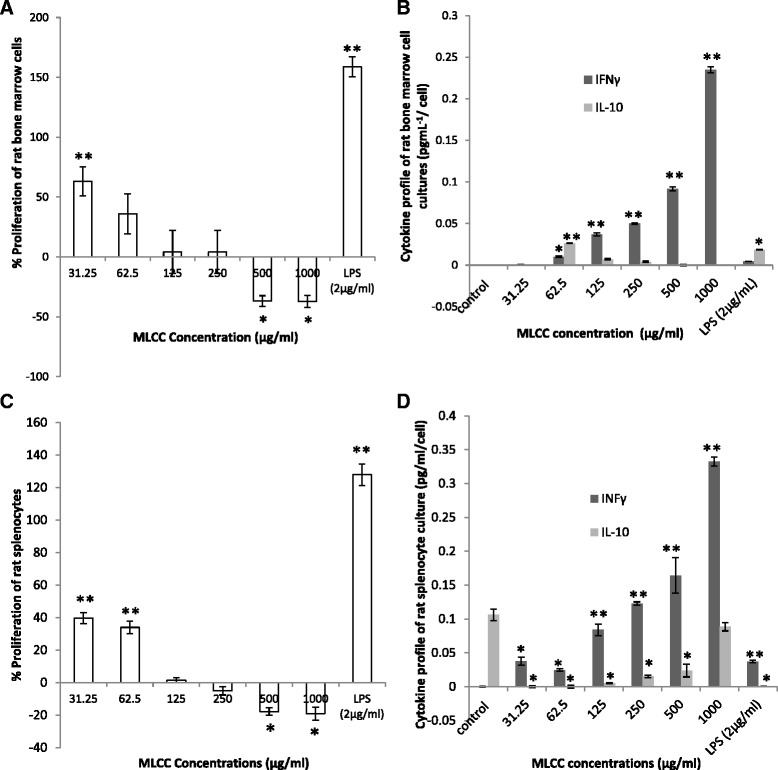
Ex vivo proliferation of rat immune cells: bone marrow cells and splenocytes treated with the MLCC, **a** effect of the MLCC on proliferation of bone marrow cells. **b** effect of the MLCC on cytokine profile of the bone marrow cells, **c** effect of the MLCC on proliferation of splenocytes. **d** effect of the MLCC on cytokine profile of the Results are expressed as Mean ± SEM (*n* = 3/concentration). *p < 0.05, **p < 0.01 were considered significant when compared with the control (ANOVA)

Compared with the control the MLCC at 31.25 μg/ml concentration significantly increased the proliferation of BMCs by 63.2% in the absence of mitogens (*p* < 0.01). Conversely, the MLCC at high concentrations of 500 and 1000 μg/ml exerted a significant cytotoxic effect with 36.76 and 37.09% cell inhibition of BMCs (*p* < 0.05), respectively (Fig. 5a).

Similarly, significant cell proliferation of 39.62 and 33.96% was observed in SC cultures treated with MLCC at 31.25 and 62.5 μg/ml concentrations, respectively (*P* < 0.01). Similar to that of the BMCs the highest tested concentrations of 500 and 1000 μg/ml manifested 17.82% and 19.08% cell inhibition respectively, indicative of significant cytotoxic activity (*p* < 0.05) (Fig. 5c).

The MLCC at 125 and 250 μg/ml concentrations had no significant effect on both BMC and SC and the cell viabilities were comparable to that of the control.

LPS (2 μg/ml), a known mitogen, elicited a significant enhancement of both BMC (158.8%) and SC (127.88%) proliferation, respectively as presented in Figs. 5a, c (*p* < 0.01).

#### Cytokine profile of rat BMC and SC culture supernatants

IFNγ (Th1 cytokine) levels of BMCs treated with MLCC with the exception of 31.25 μg/ml concentration were significantly higher compared to that of the control (Fig. 5b) (*p* < 0.05). However, IL-10 level of BMCs was only increased by the 62.5 μg/ml MLCC treatment (*p* < 0.05) (Fig. 5b).

All tested concentrations of the MLCC significantly stimulated the secretion of IFNγ from the SCs compared with the control (*p* < 0.05). Conversely, the MLCC at 31.25, 62.5 and 125, 250 and 500 μg/ml concentrations significantly inhibited the secretion of IL-10 from SCs compared with the control (*p* < 0.05) (Fig. 5d). While SCs treated with 1000 μg/ml was comparable to that of the control (*p* > 0.05) (Fig. 5d).

The BMCs treated with LPS (2 μg/ml) significantly increased the IL-10 (*p* < 0.05) level while IFNγ was comparable (*p* > 0.05) to that of the control. Conversely, SCs treated with LPS significantly elevated IFNγ level and inhibited the IL-10 level compared to that of the control (*p* < 0.05) (Figs. 5b, d).

### Acute toxicity of the MLCC on rats

**Fig. 6:**
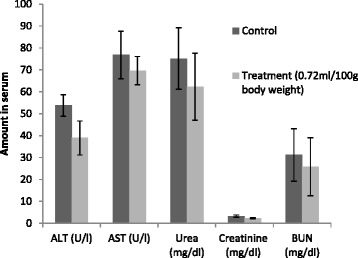
Effect of the oral administration of the MLCC on hepato (AST,ALT) and renal parameters (urea, BUN and creatinine) of rats. Results are expressed as Mean ± SEM for the number of animals used (*n* = 6/group). *p < 0.05 were considered significant when compared with the control (Mann Whitney U test)

Acute oral treatment of the highest dose (0.72 ml/100g BW) of the MLCC elicited neither hepato nor renal toxicities. Serum levels of liver functional parameters (ALT and AST) were not significantly different in the treated group compared to the control (*p* > 0.05) (Fig. 6). Similarly, renal parameters (urea, BUN and creatinine) were unaltered in the test group compared to the control (Fig. 6).

### Chemical profile of the MLCC

**Fig. 7:**
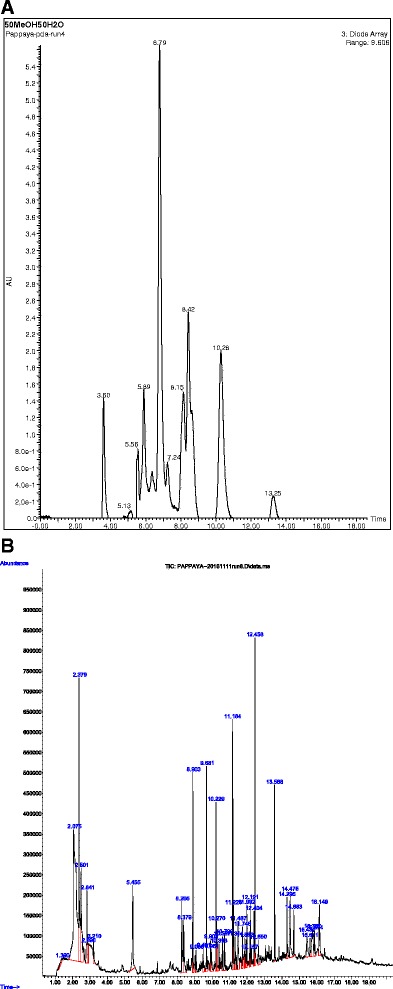
Chemical Profiling of the MLCC. **a** LC-MS chromatogram, **b** GC-MS chromatogram of the MLCC. Molecular weights, peak retention times and wave length of the peaks are given

Figure 7a represents the major peaks obtained for the MLCC from the PDA detector. Retention times and wavelengths of major peaks observed are summarised in Table 1.

**Table 1:** Retention times and the wavelengths of the compounds of the MLCC detected by the LC-MS

Peak Retention time (min)	Wave length (nm)
3.60	489
5.13	252
5.56	263
5.89	280
6.35	280
6.79	328
7.24	245
8.15	314
8.42	316
10.28	343
13.25	258

The GC-MS chromatographic peaks are presented in Fig. 7b and the identified compounds are lists in Table 2.

**Table 2:** Chemical compounds of the MLCC identified from GC-MS

No	t_R_	m/z	Assignment
1	1.390	260.9, 202.1, 157.1, 103.9, 63.2	Bis (2-(2-chloroethoxy)ethyl) ether
2	2.075	369.9, 338, 105.1, 75	Dimethoxydimethylsilane
3	2.379	280.9, 241, 207.2, 91, 61	3-Benzoyl-8-oxo-6-azabicyclo[3.2.1]octan-6,7-dicarboxylicacid, dibenzyl ester
4	2.501	281, 265, 105, 91, 77	Benzhydrazide
5	2.841	233.1, 206.9, 87.1, 61	o-Butylisourea
6	2.890	267.1, 210, 193, 87.1, 55.1	10-Oxatetracyclo[5.5.2.0(1,5).0(8,12)]tetradecene-9,11,14-trione, 4-[(2-methoxyethoxy)methoxyl]-5-methyl-
7	3.210	206.8, 190.9, 107, 91.1, 77	2-chloro-5,5-dimethyl-1-phenyl-3-hexen-1-ol
8	5.455	207, 179, 163, 119	2-methoxybenzeneacetaldehyde
9	8.266		1-methyl-2-pyrrolidinone
10	8.379	280.9, 253.1, 223	Benzonitrile
11	8.903	206.9, 142.1, 98.1, 82.1	Nonanal
12	9.086	355.1, 281.1, 207, 119.1	Octanoic acid, methyl ester
13	9.491	355.9, 207.1, 119.1	Octanoic acid
14	9.681	280.8, 208, 168.1, 128	1-Decene
15	9.903	281.1, 266.8, 207, 120.1	n-benzyl-n-phenylethylisobutyramide
16	9.951	370.8, 280.9, 207, 74.1	Nonanoic acid, methyl ester
17	10.229	207, 190.2, 175.1, 91	Benzene, 1,3-bis(1,1-dimethylethyl)-
18	10.270	206.8, 181, 158.2, 73.1	Nonanoic acid
19	10.393	206.9, 169.1, 148, 85.1, 71.1	1-Iodooctadecane
20	10.587	206.9, 167.9, 155.1, 142.1	2-Methylnaphthalene
21	10.720	429.1, 341, 281.1, 207	2-Methylnaphthalene
22	11.184	401.1, 354.9, 267.1, 196.2	2-Tetradecene
23	11.226	355, 326.8, 297, 144,.1	10-Undecenoic acid, methyl ester
24	11.314	207.1, 189, 152, 82	Dodecanal
25	11.487	280.9, 207, 192.1, 156	1,4-dimethylnaphthalene
26	11.748	327.1, 281, 206.9, 144.1	9-Oxononanoic acid
27	11.882	207, 168.1, 141, 113.2	1-Hentriacontane
28	11.992	280.9, 206.1, 191.2, 175.1	2,4-Di-tert-butylphenol
29	12.157	291.2, 207.1, 182.2, 155	1-Iodooctadecane
30	12.191	280.9, 207.1, 185.1, 152	Nonanedioic acid, dimethyl ester
31	12.404	283.1, 252.9, 207.1, 152.1	Azelaic acid
32	12.456	280.9, 224.2, 196.2, 97.1	2-Tetradecene
33	12.650	355, 281.2, 207.1, 152.1	Azelaic acid
34	13.588	281, 252.2, 224.1, 97.1	1-Octadecene
35	14.295	355.2, 326.8, 270.2, 227.1	1-Hexadecanoic acid
36	14.475	405.2, 355, 328, 256.3	n- Hexadecanoic acid
37	14.683	405, 355.1, 327.1, 280.9	Cycloeicosane
38	15.437	405.1, 355, 331, 281	9-Octadecenoic acid, methyl ester
39	15.611	355, 327, 298.3, 281	Methyl Stearate
40	15.757	405.1, 355, 326.9, 294.2	Methyl 2-octylcyclopropene-1-heptanoate
41	16.148	429.1405.1, 355.2, 294.1	9,12-Octadecadienoic acid

## Discussion

The present study for the first time established, that the mature leaf concentrate of the Sri Lankan wild type cultivar of *C. papaya* modulates both nonfunctional and functional immune responses of Wistar rats.

The oral administration of MLCC for 3 consecutive days had significantly ameliorated rat immune cell counts* i.e.* platelets, WBC and BMCs, where platelet increasing activity was pronounced. Increase of both platelets and WBCs were consistent with previous studies established with non-thrombocytopenic [14, 15] as well as thrombocytopenic murine models [12, 13].

The immunostimulatory potential of the MLCC is well illustrated in the numerical increment of total WBCs as well as of sub populations of monocytes and lymphocytes. Significant increase in the percentage of circulating mononuclear cells (lymphocytes and monocytes) indicated the effectiveness of the MLCC on both innate and adaptive arms of the immune system [1]. Previously, aqueous leaf extract of *C. papaya* treated peripheral blood mononuclear cells (PBMCs) elicited an up regulation of 23 genes mainly including monocyte chemo-attractant protein-1, 2 and 3 (MCP-1, MCP-2 and MCP-3) [19]. These proteins regulate the migration and infiltration of monocytes/macrophage [27]. Thus, activation of monocytes in numbers and function by the papaya leaf constituents raise the possibility of enhancing immunological surveillance.

The mechanism of the papaya leaf constituents induced platelet and leukocyte increment continues to be explored. Augmentation of bone marrow hematopoiesis is the most debated mechanism in literature [28]. Tham et al., [29] established that the leaf extract of *C. papaya* can reverse lead acetate induced oxidative damage by regeneration and stimulation of hematopoiesis, particularly of myeloblasts and megakaryocytes as evident in histopatholoigcal examination [29]. Moreover, the unaltered levels of SC counts which are mature lymphocytes also highlight the preference of the MLCC on hematopoietic stem cells. Therefore, it may be posited that the MLCC specifically stimulates hematopoietic stem cells to propagate and differentiate into precursor cells of megakaryocytes and those of leukocyte origin which will be mobilized in peripheral blood. In addition to the stimulation of BMCs, a rapid elevation of platelets in thrombocytopenic rats following oral gavage of MLCC was surmised to the release of stored platelets from the spleen [11]. An observation of platelet increment by the low dose of MLCC irrespective of significant upsurge of BMCs agrees with the probable release of stored platelets in the spleen.

Previously, upregulated synthesis of IL-6, a major thrombopoietic cytokine, was observed in human peripheral blood lymphocytes and stem cells from exfoliated deciduous teeth following the treatment with unripe papaya extract rich in papain [30]. This finding prompted us to investigate the effects of the oral gavage of MLCC on rat cytokine levels which may regulate the immune responses.

The present study established, in contrast to Aziz et al. [30], that plasma levels of IL-6 were significantly reduced in rats treated with the MLCC. Although, papaya based extracts are known to induce IL-6 levels in in vitro stem cell cultures, oral administration of the MLCC had reduced the systemic IL-6 levels. This paradox may be due to different experimental systems and pleiotropic activity of IL-6 [31].

Similar to that of IL-6, TNFα levels were significantly reduced by the oral treatment of MLCC. Conversely, the reduction of IFNγ levels was not significant. Since, IL-10 levels were lower than the detection levels of the ELISA kits used, the influence of MLCC on IL-10 cannot be predicted. However, inhibition of TNFα indicated anti-inflammatory property of the MLCC which was established previously [11, 32].

The MLCC, administered orally to rats enhanced phagocytosis of peritoneal macrophages and suggested a modulation of functional immunity of rats. Consistent with our findings, Tomar et al. [21] in 2012 demonstrated that oral treatment of an alcohol extract of *C. papaya* leaf significantly increased carbon clearance by the rat reticular-endothelial system that provided evidence for enhanced phagocytosis [21].

The in vitro phagocytic activity based on NBT dye reduction assay, revealed an enhancement of phagocytosis by the MLCC treatment. However, precise dose dependency was not observed. Increased phagocytosis in both in vitro and in vivo experiments signifies the immunostimulatory potential of the MLCC.

Increased phagocytic activity of the PMs was correlated with Th1 type cytokine response. IFNγ enhances phagocytosis and increases protection towards pathogens [33]. The MLCC mediated IL-10 may stimulate the anti-inflammatory responses [34] and may lessen the consequences of inflammation.

The functional immunological assays based on ex vivo proliferation provide more concrete evidence for immunomodulatory potential of the MLCC. The proliferation ability of the MLCC was compared with LPS, a bacterial endotoxin that revealed paradoxical activity *i.e.* low concentrations of the MLCC stimulated the proliferation while higher concentrations exhibited cytotoxic activity. Such a biphasic dose responsive effect is termed “hormesis” [35]; A comparable response pattern was observed in in vitro membrane stabilization potential of papaya leaf extract [36]. Thus, we assume bioactivities of the papaya leaf constituents may follow this type of dose response.

The significant proliferation of BMCs and SCs by the low concentration of the MLCC may be attributed to the presence of single or several types of mitogens or growth factors. Plant mitogens such as lectins are glycoproteins that nonspecifically bind to cell surfaces and stimulate cells to undergo mitosis [37]. However, these are selective in triggering T or B cell populations. Previously, several mitogens such as Phytohaemagglutinin(PHA), concanavalin A (Con A) and Pokeween Mitogen (PWM) have been identified from plants and these mostly impacted on the proliferation of T lymphocytes [37].Conversely, polysaccharides, polyneucleotides or lipoproteins from bacterial cell wall favor B cell proliferation. There is a high possibility that fresh plant extracts such as the MLCC may contain bacteria and their products (endotoxins) which may provide false positive results. To eliminate such contaminations the MLCC was filtered using 0.22 μm filter and furthermore, contamination of bacterial endotoxins was ruled out due to negative results obtained from the LAL test, confirming that the mitogenic activity indeed resulted due to phytoconstituents of the MLCC.

Previously, human PBMC cultures treated with *C. papaya* aqueous leaf extract revealed an enhanced production of cytokines, such as IL-12p40, IL-12p70, IFN-γ and TNF-α without growth inhibition [19]. A similar pattern of IFNγ release was observed in both rat cell types (BM and SC) we tested, but in contrast to Ostuski et al. [19] we observed significant cell inhibition at high concentrations (500 and 1000 μg/ml) of the MLCC. This disparity may be due to different types of preparation of the papaya leaf, different papaya cultivars used, and cell types used in these two studies.

IFNγ has been specifically reported as a hematopoietic inhibitor as it attenuates human bone marrow colony formation and inhibits CD34+ bone marrow cells [38]. Thus, IFNγ may have played a major role in the cell inhibition observed in high concentrations of the MLCC (500 and 1000 μg/ml). Similarly, elevated level of IFNγ was observed in SCs upon treatment with the MLCC and higher IFNγ levels were correlated with the inhibition of SCs counts. Thus, MLCC induced higher IFNγ may contribute to the inhibition of both BMCs and SCs under in vitro conditions.

Both in vivo and in vitro murine models verified the remarkable immunomodulatory potential of the MLCC. In vivo immunomodulation indicated that the active constituents of the MLCC are indeed bioavailable and effective against the ethno -pharmacologically accepted oral route [39].

The ethno - pharmacological significance of the MLCC was further emphasized by its safety. Though, the MLCC exerted in vitro cytotoxicity at higher concentrations against cultured cells it was well tolerated by rats showing no overt signs of toxicity, stress, aversive behavior or behavioral changes. Further, hepato and renal toxicity were also ruled out. Also, the MLCC failed to alter the body weights and the weights of vital organs of the test rats.

The chemical profile data obtained from LC-MS and GC-MS chromatograms revealed the presence of several bioactive compounds. The LC-MS profile exhibited several peaks and it is presumed these peaks resemble both mitogenic and anti-inflammatory compounds. Among them phenolics and flavonoids may be prominent as previous studies reported phenolic and flavonoids were abundant in the papaya leaf [40]. Further, studies are required to identify the compounds obtained from the LC-MS analysis.

Several potent anti-inflammatory compounds such as Azelaic acid [41], 1-Hexadecanoic acid [42], antioxidant compounds; 2,4-Di-tert-butylphenol [43], were elucidated in the GC-MS analysis. Hence, reported immunomodulatory properties of the MLCC could be attributed to these compounds. In addition, the GC-MS profile revealed the presence of several cytotoxic compounds such as 9-Octadecenoic acid methyl ester [44], 2,4-Di-tert-butylphenol [43] and Benzhydrazide [45]. Thus, these compounds may have contributed to the MLCC induced cytotoxicity observed in higher MLCC concetrations in in vitro cultures of PM, BMC and SCs and warrant the investigation of anticancer activity of the MLCC.

The remarkable ability of differential modulation of the immune system was previously reported for a few plants such as *Echinacea angustifolia* and *Pelargonium sidoides Echinacea* [39]. However some preparations are active only in in vitro systems. Nevertheless, the MLCC is an orally active, safe, and readily available preparation which has the potential to develop a therapeutic lead, with immense benefit to individuals suffering from immune disorders such as infections, autoimmune diseases and cancers.

## Conclusion

The present study in toto established that the MLCC effectively modulates the nonfunctional and functional immune responses such as stimulation of immune cell proliferation, increase of phagocytosis activity and modulates cytokine responses. Several active secondary metabolites with immunomodulatory properties were identified.

Collectively, when administered orally, the MLCC is safe (non-toxic) for a period of 3 days and is orally active, effectively modulates the immune response and inhibits pro-inflammatory cytokines which overly justify claims of traditional medicine. Hence, the MLCC may be a potential candidate for further research leading to the development of a herbal therapeutic agent for modulating the immune system in numerous diseases.

## Abbreviations

ALOX-12: Arachidonate 12-Lipoxygenase
ALT: Alanine transaminase
AST: Aspartate transaminase
BALB: Bagg Albino laboratory bred
BMC: Bone marrow cells
BUN: Blood urea nitrogen
BW: Body weight
Con A: Concanavalin A
COX: Cyclooxygenases
CRPMI 1640: Complete Roswell Park Memorial Institute
DF: Dengue fever
DHF: Dengue haemorrhagic fever
DMSO: Dimethyl sulfoxide
DW: Distilled water
EDTA: Ethylenediaminetetraacetic acid
ELISA: Enzyme Linked Immunosorbent Assay
FBS: Fetal bovine serum
GC-MS: Gas-chromatography- mass spectroscopy
HA: haemagglutination antibody
HCl: Hydrochloride acid
IFNγ: Interferon gamma
IL-10: Interleukin 10
IL-6: Interleukin 6
KOH: Pottasium hydroxide
LAL: Limulus amebocyte lysate
LC-MS: Liquid chromatography- mass spectroscopy
LOX: Lipooxygenases
LPS: Lipopolysaccharides
MAPKs: Mitogen-activated protein kinases
MCH: Mean cell haemoglobin
MCP: Monocyte chemo-attractant protein
MCV: Mean corpuscular volume
MLCC: Mature leaf concentrate of *Carica papaya*
MSD: Mass Selective Detector
MTT: 4,5-dimethylthiazole-2-yl)-2,5-diphenyltetrazolium bromide
NBT: Nitrobluetetrazolium, NIST- National Institute of standards and Technology (NIST)
OECD: Organization for Economic Co-operation and Development
PBMC: Peripheral blood mononuclear cells
PBS: Phosphate Buffered Saline
PDA: photodiode array
PHA: Phytohaemagglutinin
PMs: Peritoneal macrophages
PWM: Pokeween Mitogen
RPMI 1640: Roswell Park Memorial Institute 1640
SC: Splenocytes
SEM: Standard error of means
Th cells: T helper cells
TNF α: Tumor Necrosis Factor α
WBC: white blood cells
